# Detection of cervical precancerous lesions and cancer by small-scale RT-qPCR analysis of oppositely deregulated mRNAs pairs in cytological smears

**DOI:** 10.3389/fonc.2024.1491737

**Published:** 2025-01-07

**Authors:** Anastasia A. Artyukh, Mikhail K. Ivanov, Sergei E. Titov, Victoria V. Dzyubenko, Sergey E. Krasilnikov, Anastasia O. Shumeikina, Nikita A. Afanasev, Anastasia V. Malek, Sergei A. Glushkov, Eduard F. Agletdinov

**Affiliations:** ^1^ AO Vector-Best, Novosibirsk, Russia; ^2^ Department of the Structure and Function of Chromosomes, Institute of Molecular and Cellular Biology, Siberian Branch of Russian Academy of Sciences, Novosibirsk, Russia; ^3^ Federal State Budget Scientific Institution "Federal Research Center of Fundamental and Translational Medicine", Novosibirsk, Russia; ^4^ Department of Obstetrics and Gynecology, Novosibirsk State University, Novosibirsk, Russia; ^5^ Institute of Oncology and Neurosurgery, E. Meshalkin National Medical Research Center, Novosibirsk, Russia; ^6^ Department of Cervical Pathology, Saint-Petersburg City Clinic №17, Saint-Petersburg, Russia; ^7^ Subcellular Technology Lab, N.N. Petrov National Medical Research Center of Oncology, Saint Petersburg, Russia

**Keywords:** cervical cancer, squamous intraepithelial lesion, cervical screening, cytological smear, molecular biomarker, cellular mRNA, *CDKN2A*, RT-qPCR

## Abstract

**Background:**

Cervical screening, aimed at detecting precancerous lesions and preventing cancer, is based on cytology and HPV testing. Both methods have limitations, the main ones being the variable diagnostic sensitivity of cytology and the moderate specificity of HPV testing. Various molecular biomarkers are proposed in recent years to improve cervical cancer management, including a number of mRNAs encoded by human genes involved in carcinogenesis. Many scientific papers have shown that the expression patterns of cellular mRNAs reflect the severity of the lesion, and their analysis in cervical smears may outperform HPV testing in terms of diagnostic specificity. However, such analysis has not yet been implemented in broad clinical practice. Our aim was to devise an assay detecting severe cervical lesions (≥HSIL) via analysis of cellular mRNA expression in cytological smears.

**Methods:**

Through logistic regression analysis of a reverse-transcription quantitative PCR (RT-qPCR) dataset generated from analysis of six mRNAs in 167 cervical smears with various cytological diagnoses, we generated a family of linear classifiers based on paired mRNA concentration ratios. Each classifier outputs a dimensionless decision function (DF) value that increases with lesion severity. Additionally, in the same specimens, the HPV genotyping, viral load assessment, diagnosis of cervicovaginal microbiome imbalance and profiling of some relevant mRNAs and miRNAs were performed by qPCR-based methods.

**Results:**

The best classifiers were obtained with pairs of mRNAs whose expression changes in opposite directions during lesion progression. With this approach based on a five-mRNA combination (*CDKN2A*, *MAL*, *TMPRSS4*, *CRNN*, and *ECM1*), we generated a classifier having ROC AUC 0.935, diagnostic sensitivity 89.7%, and specificity 87.6% for ≥HSIL detection. Based on this classifier, a two-tube RT-qPCR based assay was developed and it confirmed the preliminary characteristics on 120 cervical smears from the test sample. DF values weakly correlated with HPV loads and cervicovaginal microbiome imbalance, thus being independent markers of ≥HSIL risk.

**Conclusion:**

Thus, we propose a high-throughput method for detecting ≥HSIL cervical lesions by RT-qPCR analysis of several cellular mRNAs. The method is suitable for the analysis of cervical cytological smears prepared by a routine method. Further clinical validation is necessary to clarify its clinical potential.

## 1 Introduction

Cervical cancer is preventable if detected at early stages of progression and treated timely. Nonetheless, it remains one of leading causes of morbidity and mortality among women. This problem can largely be attributed to shortcomings of disease prevention methods. Vaccination against high-risk human papillomavirus (HR-HPV) types (which are the main etiological factor for most of cervical lesions) covers only the most common types of HPV and is not generally available in most countries. Cervical screening, intended to detect the disease at early stages and to predict risks, is based on methods that have considerable drawbacks. In particular, cytological examination of cervical scrapings (cytology), which is the main technique for primary screening, has moderate and variable diagnostic sensitivity (~30% to 90% for high-grade squamous intraepithelial lesion or later stage, ≥HSIL) and low reproducibility of repeated tests (1–5), and consequently approximately half of severe lesions go undetected. The above characteristics are influenced by many factors: qualifications and experience of the cytologist, the organization of the laboratory, the technique and quality of sampling, and fixation and staining of cytological material (6). Besides, diagnoses based on cellular morphology are subjective and sometimes even experienced specialists disagree with each other (7, 8). HPV testing—combined with cytology or used instead of it in primary screening—has widely ranging diagnostic specificity (~40% to 98% for ≥HSIL) (9–13) due to the high percentage of transient infections (~90% are eliminated within 2 years) (14), varied prevalence of papillomavirus infections in different populations and age groups (5), and different types of employed assays. These issues lead to overdiagnosis and numerous unnecessary referrals of women for colposcopy. Moreover, such methods are unable to detect HPV-negative cases of severe cervical lesions progressing along an HPV-independent pathway as well as cases associated with atypical HPV genotypes (together they can account for up to 15% of cases, and if we take adenocarcinomas separately, then up to 38%) (15, 16). Furthermore, as HR-HPV vaccination programs are implemented, the contribution of such cases to the proportion of false negative screening results will increase.

In this regard, a search is underway for improving the diagnosis of cervical lesions by using additional molecular biomarkers. Promising solutions include the use of biomarkers of various types, such as proteins; methylation of viral or cellular DNA; cellular mRNAs, microRNAs (miRNAs, miRs), circular RNAs, or long noncoding RNAs; and cervical microbiome composition (17–23). Some of them may be useful for early detection of the disease and/or for triaging HPV-positive patients by weighing the risk of cervical pathology, and others can serve as supporting tools for better prognosis or for molecular typing of a lesion or choosing the type of treatment and monitoring its effectiveness. However, it is important to note that using combinations of different types of molecular biomarkers in a single pipeline can be problematic for routine analysis due to individual workflow requirements, starting from the preanalytical stage. Compared with a variety of cytomorphological features, which show various degrees of poor interobserver concordance (3), molecular biomarker analysis may be more objective and reproducible (24). In addition, molecular testing can be performed using high-throughput methods such as PCR, which can be useful in the face of a shortage of experienced cytologists.

This and our previous articles (25, 26) are devoted to the development of a molecular test for cervical screening that is based on the analysis of cellular biomarker RNAs in cytological smears. The approach is designed to subdivide patients into two groups: i) low-grade squamous intraepithelial lesions or earlier stages, ≤LSIL (i.e., low risk of ≥HSIL), and ii) ≥HSIL (increased risk of ≥HSIL). The division is based on fundamentally different clinical guidelines for LSILs and HSILs: while some patients require only follow-up, others require immediate in-depth examination and treatment. This is because cervical precancerous lesions have different prognoses: probabilities of regression or progression. Therefore, our aim was to design a tool that would be useful for clinicians both for identifying severe cervical lesions and for risk stratification in cases of borderline controversial conditions. Important criteria for the creation of the test (making it suitable for routine screening) were high diagnostic performance and throughput, ease of operation, and an acceptable cost. Therefore, in this work, we selected a small set of up- and down-regulated cervical-cancer–related mRNAs and attempted to use the approach (involving pairs of oppositely deregulated biomarkers) that we have previously successfully used for miRNA normalization (25). The use of paired combinations of biomarkers with different directions of expression change (instead of their ‘classical’ normalization to housekeeping genes) can minimize the number of analyzed targets in the test and maximize its diagnostic characteristics because only diagnostically useful RNAs are chosen.

## 2 Materials and methods

### 2.1 Clinical material

**Figure 1:**
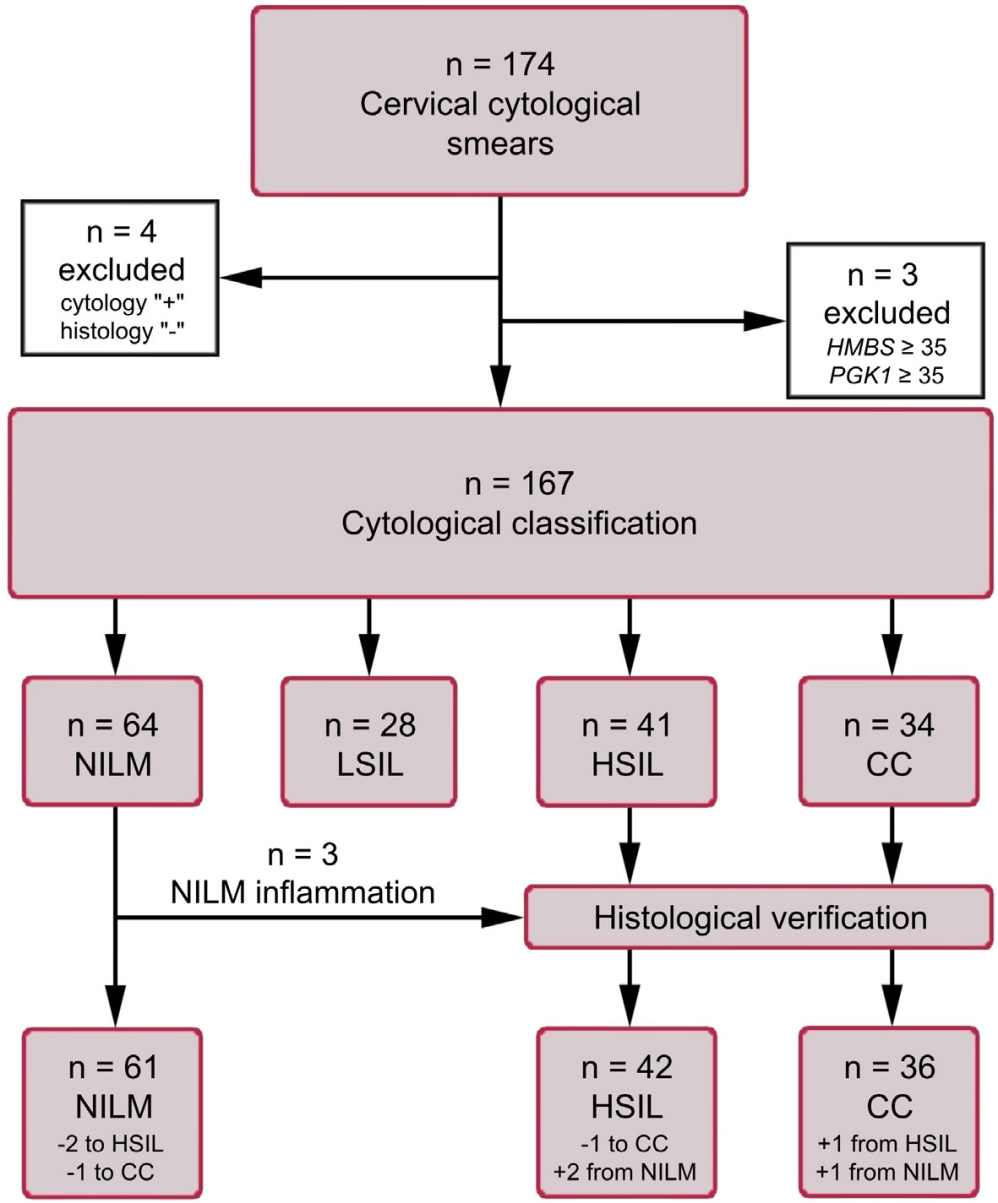
The flow diagram of the study population. *HMBS* and *PGK1* analyses were performed to assess the amount of DNA and mRNA in samples (see subsection 2.3).

Air-dried cytological smears of cervical epithelium were prepared by routine Papanicolaou staining. The material was collected from patients undergoing cytological and histological examination and treatment in oncogynecology departments of three medical centers in Russia (N.N. Petrov National Medical Research Center of Oncology, St. Petersburg; Medsanchast-168, Novosibirsk; and Novosibirsk Regional Oncology Dispensary). The cytological smears were classified according to the Bethesda system: negative for intraepithelial lesion or malignancy (NILM), LSIL, HSIL, or cervical cancer (CC). All HSIL and CC diagnoses were verified histologically. Samples with ASCUS diagnoses were intentionally excluded from the study in order to minimize their influence on the selection of biomarkers and training the algorithm for their interpretation. This is due to the fact that, in our opinion, this group of diagnoses is most susceptible to the influence of the human factor and subjectivity. The cytological smears were obtained from patients aged 24 to 81 years: NILM (n = 61, mean age 38), LSIL (n = 28, mean age 35), HSIL (n = 42, mean age 40), and CC (n = 36, mean age 44). Information about the samples is presented as a flow diagram in **Figure 1**. This study was approved by the Ethics Committee of federal government-funded institution N.N. Petrov National Medical Research Center of Oncology (extract 11/31 from protocol №3 from 16.02.2023; Saint Petersburg, Russia). Written informed consent was provided by all patients involved in the study, and the clinical data were depersonalized.

The HeLa cell line was also used, to assay the expression of mRNAs included in the prototype test and to compare it with expression levels in cytological smears that were ≥HSIL. The cell line was obtained from the cell culture collection at the State Research Center of Virology and Biotechnology VECTOR (Koltsovo, Russia).

### 2.2 Criteria for inclusion and exclusion of patients

Criteria for inclusion of patients:

1. Patients aged 21-75 years;
2. The interval for taking a sample is 5-23 days of the menstrual cycle;
3. Availability of written, personally signed, and dated voluntary informed consent to participate in the study;
4. The presence of a cytological conclusion; histological confirmation for ≥HSIL cases.

Criteria for exclusion of patients:

1. Refusal of the patient to participate in the study;
2. Pregnancy and breastfeeding period;
3. Taking a smear from the cervix within less than 1 month before the start of the study;
4. Lack of histological confirmation of ≥HSIL cytological findings;
5. Cytological conclusion other than NILM, LSIL, HSIL, CC;
6. Prior therapy for precancerous lesions and cervical cancer, endometrial cancer;
7. Any other cancer history.

### 2.3 Nucleic acid extraction

Total-nucleic-acid extraction from cytological smears and from the HeLa cell line was performed as described elsewhere (26). To assess the integrity of human DNA and mRNA in the samples, the copy number of a conserved region of the *HMBS* gene and the mRNA of housekeeping gene *PGK1* were analyzed by quantitative PCR (qPCR) and reverse-transcription quantitative PCR (RT-qPCR), respectively (primer sequences are given in **Supplementary Table S1**). Samples with an insufficient amount of human DNA (C_q_ for *HMBS* ≥ 35) or of human RNA (C_q_ for *PGK1* mRNA ≥ 35) were excluded from further analyses.

### 2.4 Selection of biomarker mRNAs

In this paper, mRNAs known to be downregulated in cervical lesions were selected, to be combined into pairs with upregulated mRNAs chosen based on our previous work (26). To do this, a systematic literature search was conducted in PubMed. We selected mRNA-coding genes for whose downregulation a statistically significant association has been reported (in relation to cervical disease severity) in at least three publications. Preference was given to studies with larger sample sizes in which clinical relevance of biomarkers was confirmed by RT-qPCR-based methods. The initial list consisted of 20 mRNA-coding genes: *ALOX12*, *CDH1*, *CFD*, *CRCT1*, *CRISP3*, *CRNN*, *CWH43*, *DSG1*, *EDN3*, *ESR1*, *HOPX*, *KRT1*, *KRT10*, *KRT13*, *KRT4*, *KRTDAP*, *MAL*, *PER1*, *PPP1R3C*, and *SPINK5*. Of these, the six most common among studies (≥ 5 publications) were selected that i) have a sufficiently high level of initial expression in cells of the normal epithelium (according to proteinatlas.org) and ii) are not downregulated in endometriosis and endometrial cancer (to exclude false positive results if such cells accidentally get into a cytological smear): *CDH1*, *PER1*, *MAL*, *CRNN*, *CRISP3*, and *SPINK5*. Their expression was assessed by RT-qPCR in a small set of cervical specimens cytologically corresponding to NILM or CC. For four of the six mRNAs (*MAL*, *CRNN*, *CRISP3*, and *SPINK5*), considerable CC-associated downregulation was confirmed, and they were chosen for further analysis along with several biomarker mRNAs from our previous work (*CDKN2A*, *TMPRSS4* [= *TSP4*], *ECM1*, and *TOP2A*).

### 2.5 Molecular analyses and data normalization

All amplification procedures were performed using a CFX96 thermal cycler (Bio-Rad Laboratories, Hercules, CA, USA). Identification, genotyping, and quantification of HR-HPV viral DNA were performed with the RealBest DNA HPV HR genotype quantitative (Cat. № D-8478, AO Vector-Best), according to the manufacturer’s instructions. The kit is designed to detect and quantify 12 HR-HPV types (HPV 16, 18, 31, 33, 35, 39, 45, 51, 52, 56, 58, and 59). Four cytological smears with signs of neoplastic lesions, identified as HPV-negative, were additionally tested for HPV DNA genotypes 26, 53, 66, 68, 73, and 82 using the RealBest DNA HPV 26/53/66 Kit and RealBest DNA HPV 68/73/82 Kit (Cat. № D-8449 and D-8451, AO Vector-Best). The viral DNA loads were estimated by the 2^-ΔCq^ method (27), using C_q_ values from amplification of the *HMBS* gene (as a marker reflecting human DNA amount) as a normalizing factor. To determine the relative enrichment of lactobacilli (LBs) and the proportion of typical opportunistic species (aerobic and anaerobic) in the total bacterial population, the RealBest BioFlor Kit (Cat. № D-4225, AO Vector-Best) was used.

For the selected six mRNAs, oligonucleotides for RT-qPCR were designed; their sequences are given in **Supplementary Table S1**. The RT-qPCR conditions were identical to those described elsewhere (26); each clinical sample was analyzed as a single replicate. Samples with C_q_ > 40 were considered expression-negative. The concentration of each biomarker mRNA was assessed semiquantitatively via normalization to housekeeping *PGK1* mRNA by the 2^-ΔCq^ method.

During the training of the linear classifiers, for each mRNA pair (mRNA_X_; mRNA_Y_), a normalized ΔC_q_ value was utilized, calculated according to the formula:


$$\Delta Cq_{X,\;Y}=\log_{2}\frac{C(mRNA_{X})}{C(mRNA_{Y})}=Cq\left(mRNA_{Y}\right)-Cq\left(mRNA_{X}\right)$$

where C(mRNA_X_) is the concentration of gene X’s mRNA, C(mRNA_Y_) is the concentration of gene Y’s mRNA, and C_q_(mRNA_X_) and C_q_(mRNA_Y_) are quantification PCR cycles for mRNAs of genes X and Y, respectively.

### 2.6 Statistical analysis, construction of classifiers, and assessment of diagnostic performance

Data were analyzed using the SciPy library of the Python programming language (28, 29). All possible paired mRNA combinations were generated to train classifiers based on them. The significance of detected differences between groups of patients was assessed by the Mann–Whitney U test in the STATISTICA v10.0 software (StatSoft, Tulsa, OK, USA). At a p-value of < 0.05, differences were considered statistically significant. To avoid the type I error, the Bonferroni correction was applied. To evaluate diagnostic characteristics (ability to detect ≥HSIL) of individual mRNAs, of biomarker pairs, and of linear classifiers, the area under the receiver-operating characteristic (ROC) curve (AUC) and diagnostic sensitivity and specificity were calculated in SPSS Statistics v23 (IBM, Armonk, New York, USA), with clinical reports of cytological findings as a reference (in cases where a histological diagnosis determined for cytological smears differed from their cytological diagnosis, the former was employed as a reference). For the best classifiers, sensitivity and specificity were calculated in five combinations for five diagnostic strategies that we tentatively selected: 1) the basic version (sensitivity ≈ specificity), 2) increased sensitivity (> 90%) with acceptable specificity (≥ 80%), 3) highly sensitive version (sensitivity 95–99%), 4) increased specificity (> 90%) with acceptable sensitivity (≥ 80%), and 5) highly specific version (specificity 95–99%). Classifiers were trained based on ΔC_q_ values for the best biomarker pairs (the lowest p-values and the best combination of diagnostic characteristics). Classification procedures were performed by means of the Python Scikit-learn library as described before (26). K-fold method was used for cross-validation. The result of applying a classifier to each cytological smear is a value of a decision function (DF) calculated using the formula:


$$w_{0}+\;\sum_{i=1}^{n}w_{i}\Delta Cq_{i}=DF$$

where ΔCq_i_ are the above-mentioned variables, w_i_ are coefficients of the variables (feature weights), n is the number of biomarker pairs, and w_0_ is an arbitrary term (the selected cutoff). DF values > 0 indicate an increased risk of ≥HSIL, which means a high probability of HSIL or CC. The distance from 0 denotes relative risks. Spearman’s correlation coefficient was calculated to assess the statistical dependence between features/data sets.

### 2.7 Extended analysis of samples for detailed examination of discordant results

The results were considered discrepant [“false positive” (FP) or “false negative” (FN) in terms of molecular classification] in cases when the “risk of ≥HSIL” based on the DF value for a cytological smear contradicted its clinical report of cytological findings. For a more detailed examination of such cases, an extended analysis was performed on 20 discrepant results of classifier 1 and on all other 147 samples with the help of additional biomarkers of different types from our previous papers (25, 26, 30): mRNAs (*ASF1B*, *KRT7*, *CD82*, *CDH3*, *TOP2A*, and *SPRR3*), miRNAs (miR-1246, miR-145, miR-196b, miR-34a, miR-20a, miR-21, miR-96, and miR-375), HPV viral load and genotypes (12 HR-HPV types: 16, 18, 31, 33, 35, 39, 45, 51, 52, 56, 58, and 59 and six additional types: 26, 53, 66, 68, 73, and 82), and a relative percentage of LBs. For RNAs, normalized values were employed: for mRNAs, normalization to housekeeping gene *PGK1* by the 2^-ΔCq^ method; and for miRNAs, normalization to miR-375 by the ΔC_q_ method. The rationale for the method of normalization of miRNAs can be found in ref (25). Based on the results of the analysis, a single heat map was constructed for all biomarkers in Microsoft Excel 2019 (Microsoft, USA).

## 3 Results

### 3.1 Diagnostic utility of selected mRNAs for the detection of ≥HSIL in cytological smears

**Figure 2:**
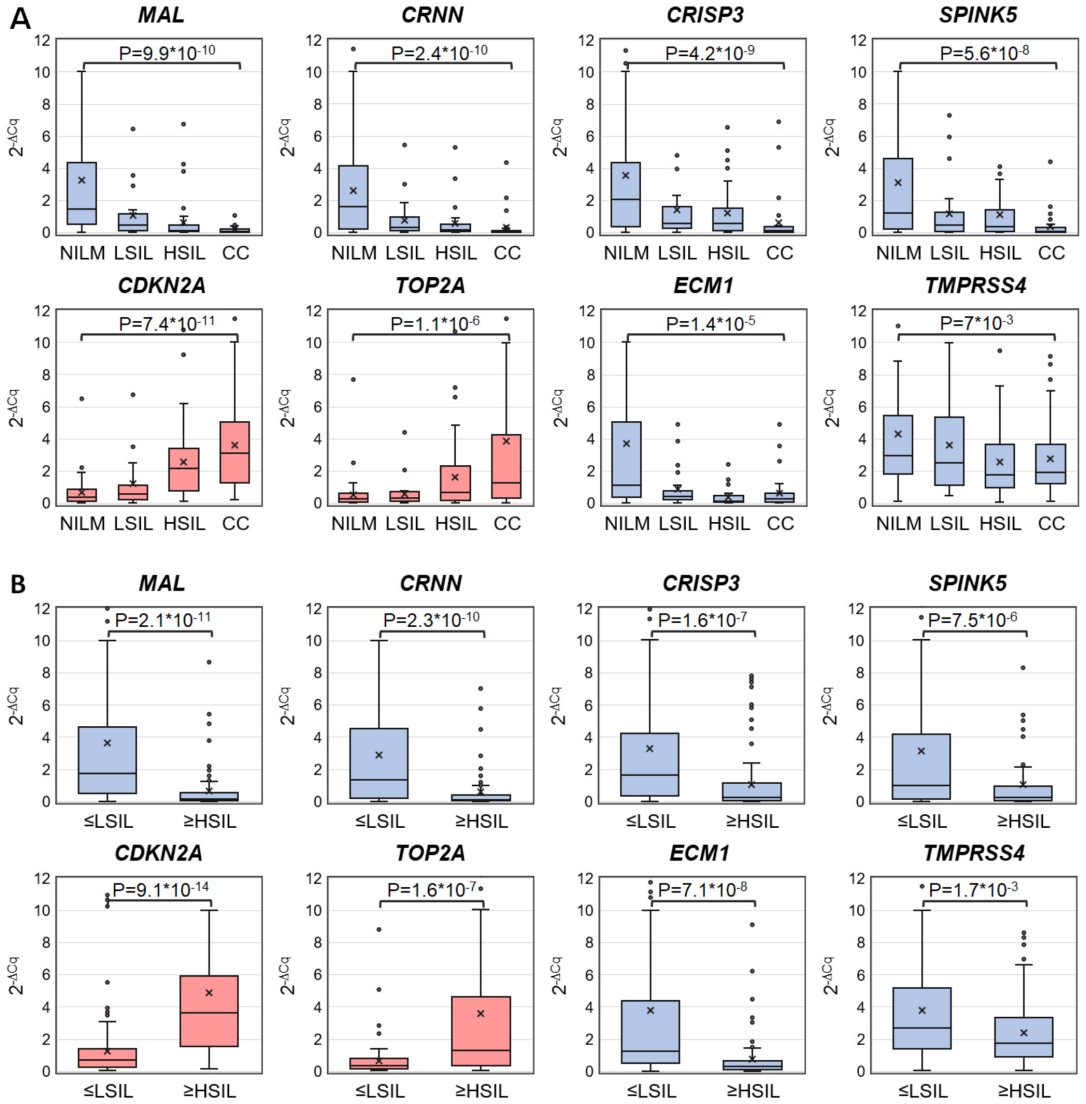
Box-whisker plots for normalized concentrations of selected mRNAs in subcategories of cervical specimens: **(A)** in different cytological diagnoses (Bethesda: NILM, LSIL, HSIL, or CC); **(B)** in combined subgroups: ≤LSIL and ≥HSIL. Blue color: downregulated mRNAs, and pink: upregulated mRNAs. 2^-ΔCq^ values are linearly transformed to bring all charts to a single scale. The charts show average values (crosses), upper and lower quartiles (boxes), median values (horizontal lines inside boxes), ranges without outliers (whiskers), and outliers (tiny circles). P-values are given above the plots.

Discriminatory power of selected mRNAs for the detection of ≥HSIL was evaluated as follows. ROC analysis—for four downregulated mRNAs (*MAL, CRNN, CRISP3*, and *SPINK5*) chosen in this study and for two upregulated (*TOP2A* and *CDKN2A*) and two downregulated (*ECM1* and *TMPRSS4*) mRNAs selected earlier (26) normalized to the housekeeping mRNA (*PGK1*)—was performed on the data obtained from the entire study population. Two classification schemes were tested for the groups formed based on cytological diagnoses: NILM versus CC and ≤LSIL versus ≥HSIL. The results are presented in **Table 1**; **Figure 2** also illustrates the distribution of relative mRNA amounts in groups of cytological smears corresponding to different diagnoses.

**Table 1:** ROC AUC and p-values for each *PGK1*-normalized mRNA, regarding the discrimination of different diagnoses.

mRNA	NILM vs CC		≤LSIL vs ≥HSIL	
	ROC AUC (95% CI)	p-value^1^	ROC AUC (95% CI)	p-value^1^
*CDKN2A*	0.882 (0.799-0.965)	7.4*10^-11^	0.825 (0.753-0.897)	9.1*10^-14^
*MAL*	0.863 (0.789-0.937)	9.9*10^-10^	0.796 (0.723-0.869)	2.1*10^-11^
*CRNN*	0.873 (0.794-0.952)	2.4*10^-10^	0.777 (0.700-0.854)	2.3*10^-10^
*ECM1*	0.739 (0.638-0.840)	1.4*10^-5^	0.732 (0.650-0.814)	7.1*10^-8^
*CRISP3*	0.847 (0.767-0.927)	4.2*10^-9^	0.728 (0.645-0.811)	1.6*10^-7^
*TOP2A*	0.778 (0.677-0.879)	1.1*10^-6^	0.725 (0.637-0.813)	1.6*10^-7^
*SPINK5*	0.824 (0.731-0.917)	5.6*10^-8^	0.694 (0.603-0.785)	7.5*10^-6^
*TMPRSS4*	0.645 (0.520-0.770)	7*10^-3^	0.650 (0.556-0.744)	1.7*10^-3^

^1^ Differences were considered significant at p < 0.05/(8*2) = 3.125*10^-3^ (taking the Bonferroni correction into account). CI, confidence interval.

Upregulated mRNA *CDKN2A* and downregulated mRNAs *MAL* and *CRNN* performed best at discriminating NILM from CC (ROC AUC > 0.85) and group ≤LSIL from ≥HSIL (ROC AUC > 0.75).

### 3.2 Diagnostic utility of mRNA pairs for the detection of ≥HSIL in cytological smears

**Figure 3:**
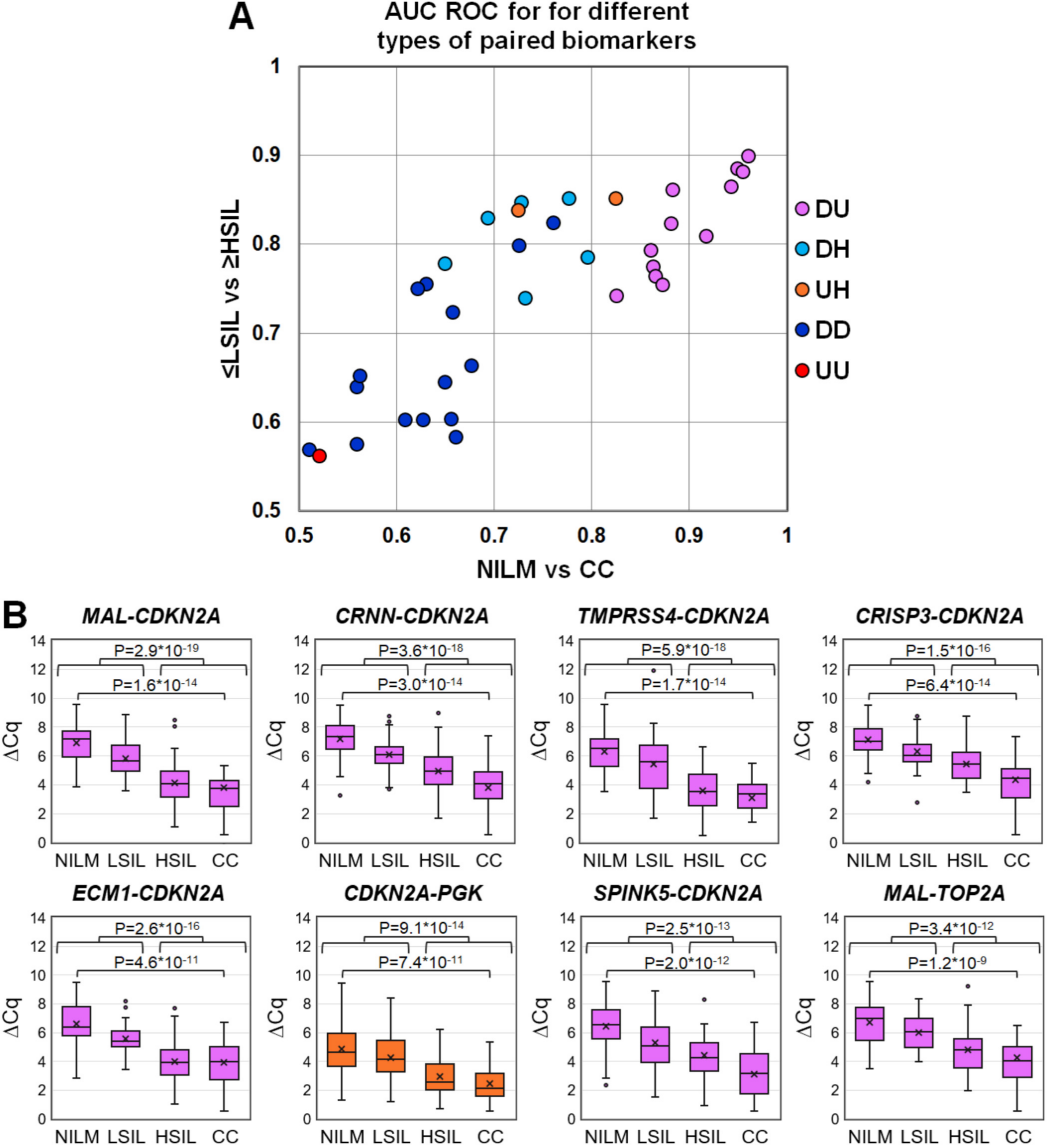
**(A)** ROC AUC values for 36 mRNA pairs in terms of discrimination of NILM from CC (x-axis) and ≤LSIL from ≥HSIL (y-axis). Colors indicate different types of mRNA pairs, combinations of up-, down-regulated, and housekeeping **(hk)** mRNAs: red, up-up (UU); dark blue, down-down (DD); orange, up-hk (UH); light blue, down-hk (DH); and pink, down-up (DU). **(B)** Box-whisker plots for ΔC_q_ values of the best mRNA pairs in different cytological diagnoses (Bethesda: NILM, LSIL, HSIL, or CC). ΔC_q_ values are linearly transformed to bring all charts to a single scale. The charts show average values (crosses), upper and lower quartiles (boxes), median values (horizontal lines inside boxes), ranges without outliers (whiskers), and outliers (tiny circles). P-values are given above the plots.

To evaluate discriminatory power of different mRNA pairs for the detection of ≥HSIL, ROC analysis for ΔC_q_ values calculated for all possible mRNA pairs (n = 36) was performed on the data obtained from the entire study population. The results are summarized in **Figure 3**, including box plots illustrating the distribution of ΔC_q_ values in groups of cytological smears corresponding to various diagnoses (all ROC AUC and p-values ​​are given in the **Supplementary Table S2**).

The pairs combining an upregulated mRNA and a downregulated mRNA performed best at discriminating NILM from CC, and five of them (*MAL-CDKN2A, CRNN-CDKN2A, TMPRSS4-CDKN2A, CRISP3-CDKN2A*, and *ECM1-CDKN2A*) also performed best at discriminating ≤LSIL from ≥HSIL as compared with the best upregulated biomarker *CDKN2A* normalized to hk-mRNA *PGK1*.

### 3.3 Diagnostic utility of different types of linear classifiers based on selected mRNA pairs for the detection of ≥HSIL in cytological smears

The diagnostic characteristics (ROC AUC, diagnostic sensitivity and specificity) of linear classifiers based on 2–6 mRNA pairs selected at the previous stage were calculated for the entire study population and compared. In **Table 2**, diagnostic characteristics of the six selected classifiers are given, as compared to the classifier from our previous work (P) (26), for which these characteristics were calculated too. The inclusion of more than four mRNA pairs into a classifier or the addition of miRNA pairs validated in ref (26). in no case considerably improved the diagnostic characteristics.

**Table 2:** Diagnostic characteristics (ROC AUC, sensitivity and specificity) of the best six linear classifiers and of the classifier from our previous work (P) (26) calculated for the entire study population.

Classifier ID	Combinations of mRNA pairs	NILM vs CC			≤LSIL vs ≥HSIL		
		ROC AUC (95% CI)	Sensitivity, % (95% CI)	Specificity, % (95% CI)	ROC AUC (95% CI)	Sensitivity, % (95% CI)	Specificity, % (95% CI)
P	*TMPRSS4-CDKN2A* *ECM1-CDKN2A* miR34a-miR375 miR96-miR375	0.961 (0.926-0.995)	86.49 (71.23-95.46)	93.44 (84.05-98.18)	0.921 (0.880-0.962)	85.90 (76.17-92.74)	86.52 (77.63-92.83)
1	*MAL-CDKN2A* *TMPRSS4-CDKN2A* *CRNN-CDKN2A* *ECM1-CDKN2A*	0.992 (0.980-1.000)	**97.30** **(85.84-99.93)**	**98.36** **(91.20-99.96)**	0.935 (0.897-0.973)	**89.74** **(80.79-95.47)**	**87.64** **(78.96-93.67)**
2	*MAL-CDKN2A* *TMPRSS4-CDKN2A* *CRNN-CDKN2A*	0.994 (0.985-1.000)	**97.30** **(85.84-99.93)**	**98.36** **(91.20-99.96)**	0.933 (0.894-0.972)	88.46 (79.22-94.59)	**87.64** **(78.96-93.67)**
3	*MAL-CDKN2A* *TMPRSS4-CDKN2A*	0.996 (0.988-1.000)	**97.30** **(85.84-99.93)**	96.72 (88.65-99.60)	0.934 (0.896-0.973)	**89.74** **(80.79-95.47)**	**87.64** **(78.96-93.67)**
4	*MAL-CDKN2A* *TMPRSS4-CDKN2A* *CRNN-CDKN2A* *CRISP3-CDKN2A*	0.994 (0.985-1.000)	**97.30** **(85.84-99.93)**	96.72 (88.65-99.60)	0.932 (0.893-0.972)	**89.74** **(80.79-95.47)**	86.52 (77.63-92.83)
5	*MAL-CDKN2A* *TMPRSS4-CDKN2A* *CRISP3-CDKN2A*	0.996 (0.988-1.000)	**97.30** **(85.84-99.93)**	96.72 (88.65-99.60)	0.932 (0.893-0.972)	**89.74** **(80.79-95.47)**	86.52 (77.63-92.83)
6	*MAL-CDKN2A* *TMPRSS4-CDKN2A* *CRISP3-CDKN2A* *CRNN-TOP2A*	0.996 (0.988-1.000)	94.59 (81.81-99.34)	96.72 (88.65-99.60)	0.931 (0.891-0.971)	88.46 (79.22-94.59)	86.52 (77.63-92.83)

The highest values ​​are highlighted in bold.

Because it is possible to obtain different combinations of sensitivity and specificity for the same test by changing the cutoffs, we additionally determined which of them could be obtained for classifiers 1–6 from **Table 2** by adjusting the cutoff for specific diagnostic strategies (described in subsection 2.6), see **Table 3**.

**Table 3:** Combinations of sensitivity (Sens) and specificity (Spec) for detection of ≥HSIL by classifiers 1–6 from **Table 2** at different cutoffs selected for four additional diagnostic strategies (described in 2.5).

Diagnostic strategies	Classifier ID											
	1		2		3		4		5		6	
	Sens, %	Spec, %	Sens, %	Spec, %	Sens, %	Spec, %	Sens, %	Spec, %	Sens, %	Spec, %	Sens, %	Spec, %
Sens > 90%, Spec ≥ 80%	91.0	82.0	91.0	79.8	**91.0**	**86.5**	91.0	80.9	91.0	82.0	91.0	78.7
Sens 95-99%	**96.2**	**68.5**	**96.2**	**68.5**	96.2	60.7	96.2	67.4	96.2	62.9	96.2	62.9
Spec > 90%, Sens ≥ 80%	**85.9**	**91.0**	**85.9**	**91.0**	80.8	92.1	**85.9**	**91.0**	83.3	91.0	85.9	91.0
Spec 95-99%	64.1	97.8	67.9	95.5	69.2	95.5	65.4	96.6	**74.4**	**95.5**	70.5	95.5

The highest values ​​are highlighted in bold.

Thus, classifier 1 was the best option for four of the five diagnostic strategies, considering that it allows the use of its shortened versions: classifier 2 (*ECM1* excluded), which had comparable diagnostic performance, and classifier 3 (*ECM1* and *CRNN* excluded), which was the best in the “Sens > 90%, Spec ≥ 80” strategy.

### 3.4 Extended analysis of samples for detailed examination of discordant results

**Figure 4:**
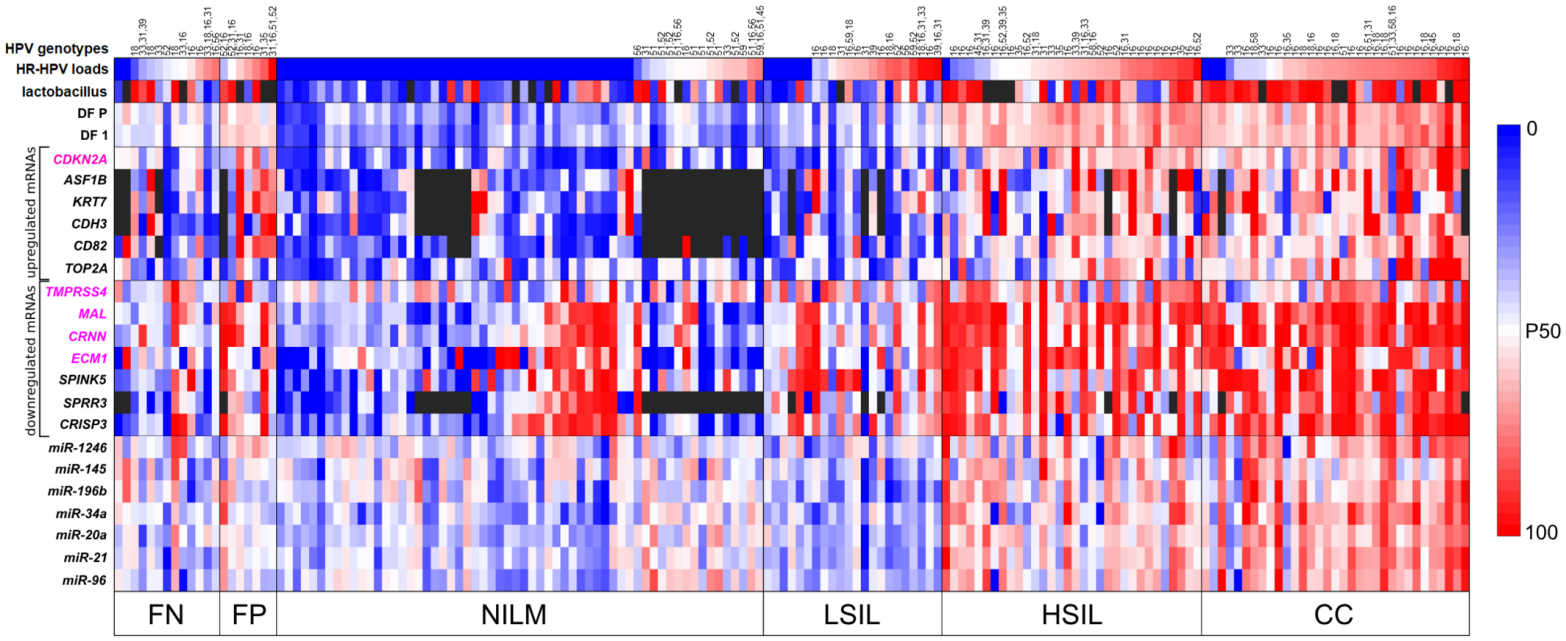
The heat map illustrating data for different types of biomarkers (HR-HPV genotypes and loads, percentage of LBs, mRNAs and miRNAs levels) for 167 cytological smears and calculated DF values of classifiers 1 and P. The samples on the map are subdivided into six groups: “false negative” (FN) and “false positive” (FP) discrepant results; NILM and LSIL identified by classifiers in group ≤LSIL (low risk of ≥HSIL); HSIL and CC identified by classifiers in group ≥HSIL (increased risk of ≥HSIL). HPV genotypes are listed in order from highest to lowest viral load. Quantitative data on each biomarker were linearly transformed to a scale from 0 (baseline expression: blue) to 100 (highly aberrant expression: red). P50 (white) denotes the 50th percentile (P50 values vary for different biomarkers). Data on downregulated mRNAs and LBs were inverted (multiplied by −1 and then added 100). mRNAs included in classifier 1 are highlighted in pink.

The results are presented as a heat map (**Figure 4**), including data on all examined biomarkers in 167 cytological smears, as well as data on classifier 1 from the current work and classifier P from the previous paper (26).

Based on the results of the analysis of additional molecular biomarkers, several types of samples were identified:

1. baseline expression (blue) for most RNAs;
2. baseline expression for most of upregulated mRNAs and miRNAs and highly aberrant expression (red) for downregulated mRNAs;
3. moderately aberrant (white) and highly aberrant expression for most RNAs;
4. highly aberrant expression for most RNAs.

In the NILM group, type 1 samples were predominant, and samples of types 2 and 3 were also present. In the LSIL group, samples of the same three types were present, but the percentage of types 2 and 3 was higher. In groups HSIL and CC, samples of type 4 predominated, and there were a few samples of types 2 and 3. In groups FN and FP, type 3 samples were predominant, with type 1 samples also present in FN, type 2 in both FN and FP, and type 4 in group FP.

**Figure 5:**
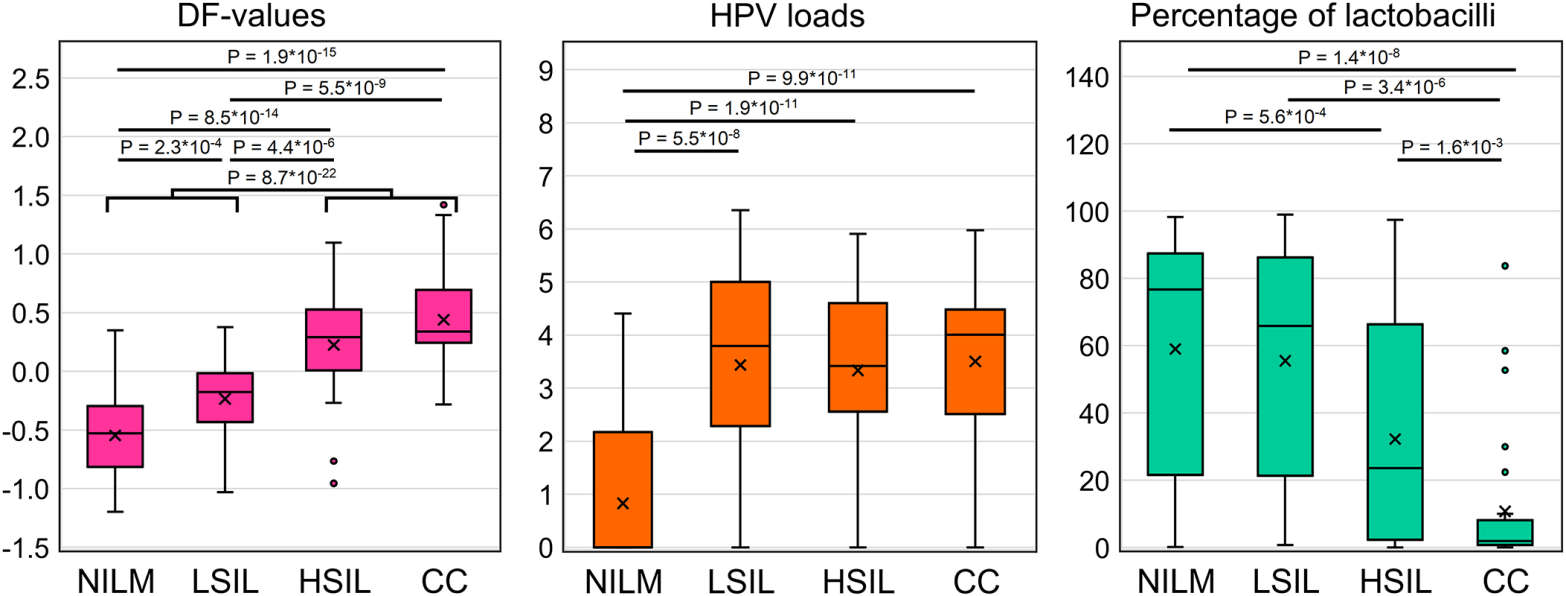
Distribution of DF values, HPV viral loads and percentage of LBs in the analyzed sample of 167 cervical cytological preparations. p-values ​​are given for comparisons for which differences were significant (p < 0.05/(7*3) = 2.4*10^-3^, with Bonferroni correction).

In total, 111 specimens from 167 (66.5%) were HR-HPV positive [NILM, 16 of 60 (26.6%); LSIL, 16 of 22 (72.7%); HSIL, 31 of 32 (96.9%); CC, 30 of 33 (90.9%); FN 11 of 13 (84.6%); FP, 7 of 7 (100%)]. In 43 of 111 (38.7%) cases multigenotype (≥2 genotypes) HPV infections were found [NILM, 7 of 16 (43.7%); LSIL, 5 of 16 (31.2%); HSIL, 10 of 31 (32.2%); CC, 11 of 30 (36.6%); FN, 4 of 11 (36.4%); FP, 6 of 7 (85.7%)]. The representation of different HR-HPV genotypes in these groups is shown in **Figure 4** and **Supplementary Table S3**. The percentage of HR-HPV-positive samples containing genotype 16 increased with the severity of cervical lesion [NILM, 3 of 16 (18.7%); LSIL, 9 of 16 (56.2%); HSIL, 21 of 31 (67.7%); CC, 24 of 30 (80%)]; and also was high in the FP group, 6 of 7 (85.7%). In addition, the NILM group showed a higher percentage of genotype 51 (68.7% of HR-HPV-positive samples) compared to other groups, where it ranged from 0 to 14%. Otherwise, no obvious associations or trends related to HPV genotypes or multigenotypic infections were found in the entire sample. Elevated viral loads (> P50) were observed in most samples from groups LSIL, HSIL, CC, and FP. Decreased LBs percentages were most common in groups HSIL and CC. The proportion of anaerobic bacterial species was inversely correlated with LBs, while no patterns were found for aerobic species (data not shown). Although in groups HSIL, CC, and FP, the values of all biomarkers mostly were moderately or highly aberrant, in groups NILM, LSIL, and FN, we did not find any obvious associations between increased HR-HPV DNA load, a decreased LBs percentage, and aberrant RNA expression. To compare the distribution of DF values, HPV loads and the percentage of LBs in the sample, box-whisker plots were constructed, see **Figure 5**. The calculation of the Spearman’s coefficient (r_s_) showed a weak correlation between the DF-values versus HPV loads and DF-values versus LBs percentage: 0.546 and ‑0.527, respectively.

### 3.5 A prototype molecular test suitable for routine use

**Figure 6:**
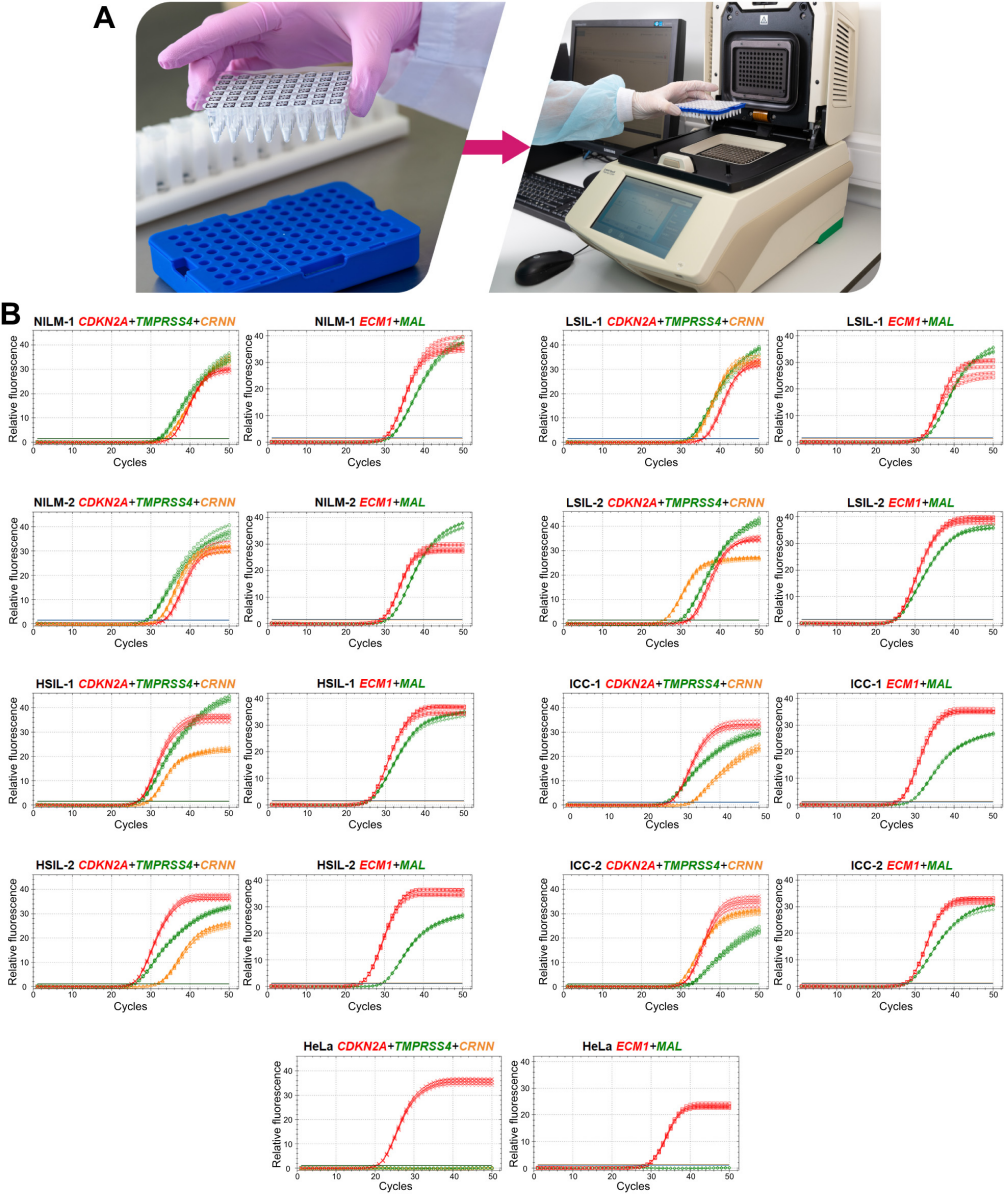
A prototype molecular test for cervical pathology detection. **(A)** To perform the test, it is necessary to reconstitute lyophilized ready-for-use PCR mixes in 50 µL of an eluate (including addition of a nucleic-acid sample: two tubes per sample), to mix and place them in a thermocycler and select an appropriate protocol of RT-qPCR. **(B)** Examples of RT-qPCR results for cervical cytological smears with different diagnoses (2 NILMs, 2 LSILs, 2 HSILs, and 2 CCs) and for cultured HeLa cells in five replicates. The colors indicate signals in different fluorescence channels: red, ROX (crosses: *CDKN2A*, squares: *ECM1*); green, FAM (circles: *TMPRSS4*, diamonds: *MAL*); orange, HEX (triangles: *CRNN*).

To devise a prototype test, the multiplex analysis of five mRNAs (providing an opportunity to use classifiers 1–3) in two tubes (1: *CDKN2A+TMPRSS4+CRNN*, 2: *MAL+ECM1*) was optimized in the format of lyophilized ready-for-use PCR mixes (**Figure 6A**). In the remaining material of 120 out of the 167 cytological smears (52 NILMs, 9 LSILs, 38 HSILs, and 21 CCs), diagnostic characteristics equivalent (sensitivity 88.14%, specificity 86.9%) to those of the nonmultiplex assay (sensitivity 88.14%, specificity 88.52%) were obtained for the same set of cytological smears. Spearman’s coefficient of correlation between DF values ​​of the two methods was high (r_s_ = 0.945). **Figure 6B** shows examples of RT-qPCR results for eight random cervical cytological smears with different diagnoses and cultured HeLa cells for comparison.

**Figure 7:**
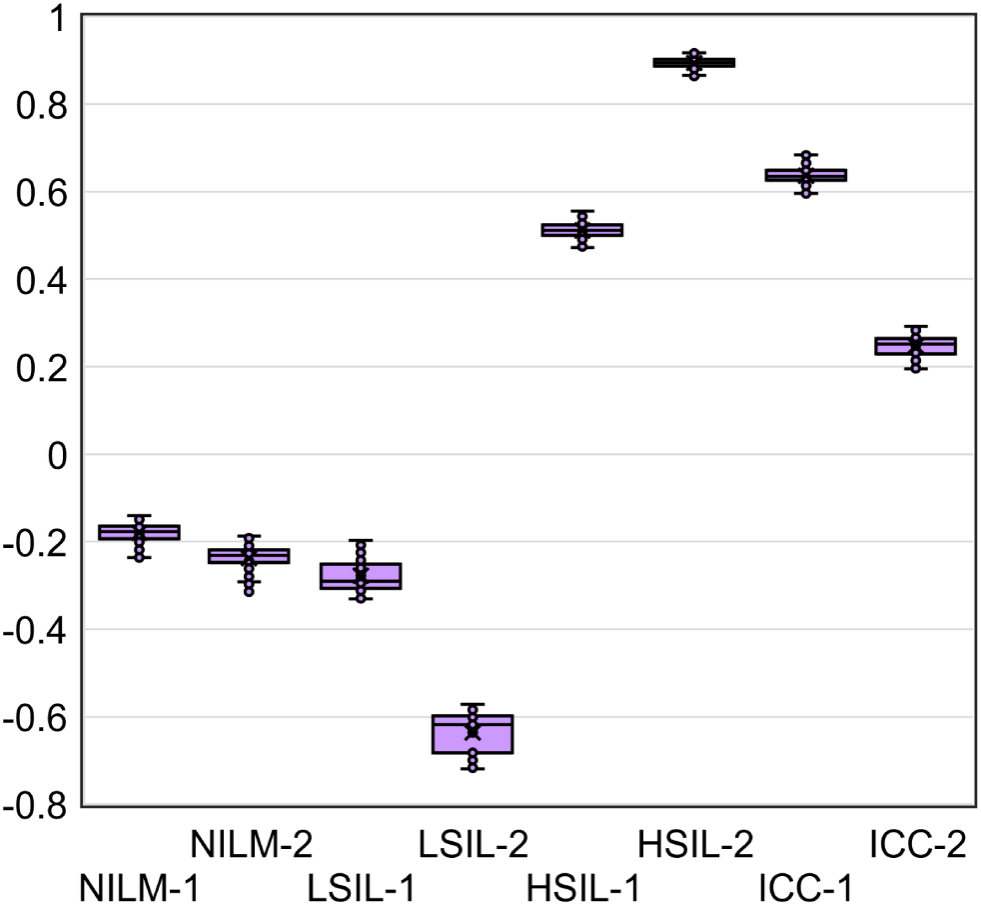
The distribution of DF values calculated from all possible combinations of C_q_ values from five RT-qPCR replicates for five mRNAs (3125 DF values for each of eight cytological smears).

Given that the accuracy of the obtained DF values partly depends on systematic error of RT-qPCR, we analyzed the samples in five replicates to estimate the contribution of this error. From the combinations of the obtained C_q_ values, all possible DF values (classifier 1) were computed (five biomarkers to the power of five replicates = 3125 DF values for each cytological smear). Their distribution for eight cytological smears is illustrated in **Figure 7** and coefficients of variation (CV) are given in **Table 4** below.

**Table 4:** Examples of the prototype test’s results for eight cervical cytological samples with different diagnoses (2 NILMs, 2 LSILs, 2 HSILs, and 2 CCs) and cultured HeLa cells: each mRNA’s C_q_ obtained from the amplification plots in **Figure 6B** and DF values calculated from them using classifier 1.

Sample ID	Average C_q_ values for each mRNA					DF values of classifier 1	CVs for DF values, %	Test results
	*CDKN2A*	*MAL*	*TMPRSS4*	*CRNN*	*ECM1*			
NILM-1	34.66	31.09	32.07	33.35	29.69	-0.1797	10.7	low risk of ≥HSIL
NILM-2	33.20	30.67	28.85	31.38	29.07	-0.2377	12.4	low risk of ≥HSIL
LSIL-1	35.57	32.36	32.02	32.75	31.34	-0.2781	12.6	low risk of ≥HSIL
LSIL-2	31.79	25.08	29.80	25.57	24.64	-0.6364	7.1	low risk of ≥HSIL
HSIL-1	26.43	25.83	27.03	29.16	25.23	0.5122	3.2	increased risk of ≥HSIL
HSIL-2	25.07	29.28	25.56	31.2	23.79	0.8935	1.2	increased risk of ≥HSIL
CC-1	27.02	28.49	26.13	32.37	25.36	0.6370	2.8	increased risk of ≥HSIL
CC-2	30.22	27.87	32.47	28.84	27.44	0.2475	8.9	increased risk of ≥HSIL
HeLa	20.44	N/A (40.00)	N/A (40.00)	N/A (40.00)	28.85	3.8160	0.3	increased risk of ≥HSIL

In cases where an amplification signal was not obtained (N/A), a C_q_ value of 40 was utilized to calculate the DF value. CV, coefficient of variation.

**Table 4** also shows the average C_q_ values, the DF values calculated from them, and the corresponding test results reflecting the risk of ≥HSIL for each of the nine samples.

The DF values’ variability associated with systematic error of RT-qPCR was low for all samples (CV ≤ 12.6%), and in none of the nine cases did it lead to overlaps with another diagnostic group.

## 4 Discussion

Every year, more and more studies come out on cellular molecular biomarkers of severe cervical lesions and cancer. These markers may improve the effectiveness of cervical screening in several ways. Firstly, they can compensate for the mediocre diagnostic sensitivity of cytology both by detecting missed severe lesions (owing to technological features of cytology and the influence of the human factor) and by identifying early molecular abnormalities that precede morphological ones and indicate a high risk of disease progression (31, 32). Secondly, the use of cellular biomarkers in combination with HPV testing provides an opportunity for risk stratification and triage to reduce the frequency of overdiagnosis and unnecessary referrals for colposcopy in case of transient infections. In addition, the analysis of cellular targets can help to detect both HPV-negative cases and those associated with infection with rare HPV genotypes not covered by the HPV tests (the prevalence of such cases will increase with the dissemination of vaccination against the main HR-HPV genotypes). A wide variety of biomarkers have already been proposed for these purposes. At present, there are a number of established tests based on different types of biomarkers: proteins: CINtec^®^ PLUS (p16 and Ki67; sensitivity 86.7%, specificity 95.2% for ≥HSIL) (33) and its analogs; methylation of cellular DNA: GynTect^®^ (*ASTN1*, *DLX1*, *ITGA4*, *RXFP3*, *SOX17*, and *ZNF671*; sensitivity 59.7%, specificity 98.0% for ≥HSIL) (34), WID^®^-can (*DPP6*, *RALYL*, and *GSX1*; sensitivity 77.0%, specificity 76.9% for ≥HSIL) (35), QIAsure Methylation Test (*FAM19A4* and *MIR124-2*; sensitivity 63.3%, specificity 67.4% for ≥HSIL) (36), and other gene panels; and miRNAs: NOVAprep^®^ miR-Cervix (miR-21, -29b, -145, -451a, -1246, and -1290; sensitivity 79.2%, specificity 79.3% for NILM vs HSIL) (37). There are no established tests for triage based on the analysis of cellular mRNAs yet; however, diagnostic utility of biomarkers of this type is stated in some studies [e.g (38–42)].

In the present work, we developed a prototype test for detection of cervical lesions at a ≥HSIL stage (increased risk of ≥HSIL) by analyzing a small set of mRNAs in cytological smears. The test (based on *CDKN2A*, *MAL*, *TMPRSS4*, *CRNN*, and *ECM1*) has characteristics comparable to those of established tests (sensitivity 89.7%, specificity 87.6% for ≥HSIL). By using pairs of oppositely deregulated mRNAs, we managed to improve diagnostic accuracy of the method compared to our previous results (26), while reducing the number of analyzed biomarkers. The sensitivity and specificity of the assay can be adjusted by changing the cutoffs. Nonetheless, the suitability of these changes—in relation to the choice of different diagnostic strategies for solving specific clinical problems—should be confirmed in further studies. The contribution of each mRNA to a classification result needs to be clarified in a larger set of cytological smears that includes a variety of diagnostic categories (including ambiguous ones such as atypical squamous cells of undetermined significance, ASCUS) to find a balance between diagnostic parameter values and the number of analyzed targets. This is also important from an economic point of view because using a smaller number of biomarkers can reduce the cost of the assay.

The proportion of discordant results in this study was 12% (20 out of 167 for classifier 1). All of them can be tentatively categorized into two groups depending on the proximity of their DF values to the classifier cutoff: Group I [4 out of 20 (20%): DF values are far from the classifier cutoff and the results on most mRNAs in the extended analysis of samples correspond to their characteristic expression in groups NILM (2 FNs) and ≥HSIL (2 FPs)] and Group II [16 out of 20 (80%): DF values are close to the classifier cutoff (within 10% of values above and below it) and the expression of most mRNAs is intermediate between (or similar to) their characteristic expression levels in groups ≤LSIL (for FNs) and ≥HSIL (for FPs)]. There are various possible reasons for such results, including

1. dilution of atypical cells in cytological smears with normal cells (FNs from groups I and II);
2. errors of the reference method: failure to detect severe lesions or overestimation of lesion severity by the cytologist owing to the human factor (FNs and FPs from groups I and II);
3. intermediate states that are difficult to interpret by any methods and probably require assignment to a gray zone (FNs and FPs from group II);
4. errors in molecular classification owing to systematic error at any stage of the analysis (FNs and FPs from group II);
5. detection of early molecular abnormalities with a high risk of progression, without pronounced morphological atypia (FPs from group II);
6. insufficient specificity of biomarkers, leading to false positive results in cases of transient infections or any other diseases or in age-related aberrations (FPs from group II);
7. special molecular subtypes that are poorly identified by selected mRNAs (FNs from group II).

Using additional molecular biomarkers, we were unable to identify specific causes of each discrepant result. Nevertheless, in cases where the results on most biomarkers were consistent, it can be assumed that the reasons for such results are not systematic error or other errors in the molecular analysis. In our view, the following may be useful in addressing this issue and facilitating decision-making:

- collecting additional information about the age of patients, their genetic characteristics, and comorbidities;
- counting of different types of cells in cytological smears (calculating the proportions of atypical and healthy cells to assess heterogeneity);
- repeating various stages of the molecular analysis;
- getting a second opinion from another cytologist on the diagnosis for the same cytological smear or a redone one.

The results of the extended analysis of the remaining 147 samples, which were identified by the molecular test consistently with their morphological diagnoses, did not allow us to confidently distinguish any clusters or molecular subclasses, partly owing to the small sample size. Nonetheless, in groups NILM and LSIL, a subgroup of samples was identified that differed from the rest: normal expression of upregulated mRNAs characteristic of ≤LSIL and aberrant expression of all downregulated mRNAs characteristic of ≥HSIL. Most of our downregulated biomarkers are associated with some specific epithelial molecular processes (e.g., epithelial differentiation, keratinization, and apical transport in polarized epithelial cells), and upregulated ones are related to the cell cycle (regulation of replication, proliferation, apoptosis, and cell adhesion). Therefore, we can suggest two possible reasons for such an expression profile. The first one is early molecular abnormalities associated with neoplasia that have an uncertain risk of progression (there is a pronounced decrease in epithelial function, but no loss of cell cycle control). The second reason is an unrelated neoplasia condition or disease that is also characterized by underexpression of the selected mRNAs (i.e., insufficient specificity of the biomarkers). Four samples with similar expression profiles were also present in ≥HSIL groups, thus possibly also indicating that this is some special “molecular subclass” or dilution of atypical cells with normal cells in the cytological smears. Another notable finding is that in ≥HSIL groups, there were cytological smears with highly aberrant expression of most biomarkers, but baseline expression of *CDKN2A*. These cytological smears may represent a subgroup of *CDKN2A*-negative lesions mentioned in ref (43). Alternatively, this result may be due to the influence of some factors on gene expression that we did not account for. The above findings confirm the necessity to use combinations of biomarkers (instead of stand-alone ones) because this approach increases the likelihood of their mutual compensation for each other’s insufficient sensitivity or specificity, including those caused by the existence of different molecular subclasses.

When regarding the results of mRNA testing (DF-values) in the context of HPV infection and cervicovaginal microbiome (CVM) condition, we observed the weak correlation between these indicators. Microbial dysbiosis is now recognized as an additional risk factor for HPV persistence and cervical neoplasia. Normally, in women of reproductive age, the CVM is dominated by LBs, which prevent infections by maintaining a weakly acidic pH, producing lysozyme, hydrogen peroxide, bacteriocins, preventing biofilm formation and having a stimulatory influence on local immunity (44, 45). The CMV dysbiosis, the most pronounced feature of which being the decrease in the concentration of LBs, is usually a consequence of weakening of either systemic or local immunity, which, in its turn, is modulated by the CVM (46, 47). Dysbiosis is accompanied by growing biodiversity and an increased risk of infections caused by various pathogens. The dysbiotic CVM raises the risk of HR-HPV infection and persistence, which in turn trigger SIL development. As the result, cervical lesions are generally accompanied by the CVM dysbiosis (23, 48, 49). In the present work, we chose as an indicator of dysbiosis the relative proportion of LBs DNA in total bacterial DNA as the most universal parameter that could be easily estimated using a simple PCR-based method. In our sample, we observed significant differences in the proportion of LBs between patients with different diagnoses. However, they did not differ significantly between NILM and LSIL, as well as between LSIL and HSIL (see **Figure 5**) and were weakly correlated with DF values. Thus, despite the fact that CVM analysis can act as a triage tool, this may require more complex methods than a simple assessment of the relative proportion of LBs. Of note is that our sample was enriched in women with no LBs dominance, which is most likely due to age-related effects.

While the possibility of using CVM analysis as a triage tool has only been discussed relatively recently, attempts to use viral load and HPV genotyping for the same purposes have a long history. As HR-HPV testing cannot differentiate productive from transforming HPV infections, does not identify HR-HPV-negative lesions, and also has a low positive predictive value (PPV) of HSIL and CC detection, researchers are trying to analyze additional parameters of HPV infection to increase PPV. Among them are viral load and genotype. Unfortunately, both are not informative enough. The high HPV viral load in older ages is considered a surrogate marker of the HPV persistence pointing to an increased risk of malignant transformation. However, although HPV viral load may be an indicator of progression of precancerous cervical lesions, this association was shown to be age-, genotype-, and population-dependent, which limits its use as a triage indicator (50, 51). Currently, so-called “partial genotyping” of HR-HPV is commonly used in cervical screening algorithms; this is specific identification of the most aggressive genotypes (HPV16 and HPV18), which implies a more conservative approach to the management of women in whom less aggressive genotypes are found (52). Nevertheless, some studies indicate that (a) “more aggressive” genotypes do not significantly differ in the probability and rate of clearance, and (b) their contribution to the incidence of cervical pathology may be affected by the vaccination programs targeting these types. In this regard, extended genotyping may be more useful for management, which, however, involves more complex interpretation algorithms that have yet to be refined (53, 54). In our study sample, the relative frequency of HPV16 increased from NILM to CC groups (see **Supplementary Table S3**), which supports the concept of partial genotyping. As for the viral loads, they were not able to discriminate LSILs, HSILs and CCs (**Figure 5**), in line with our own and other studies, and also weakly correlated with DF values among these patient subgroups. The low correlation between DF values, HR-HPV viral loads and LBs DNA percentage, allows them to be considered as separate risk factors for cervical pathology, which could complement each other in the management of patients.

Some limitations of the study should be mentioned. The first one is the small sample size, and the second one is prevalence (of samples with various diagnoses) that differs substantially from that in the real-world patient population (where the proportion of HSIL and cancer is < 4%). These shortcomings could distort the calculation of diagnostic characteristics, leading to some overfitting. Thirdly, there were no diagnoses of ASCUS in our sample; the interpretation and classification of this pathology by means of molecular tools is one of the most urgent tasks. Another possible source of bias in the training of the 428 classifiers and in the calculation of diagnostic characteristics was the lack of histological verification of NILM and LSIL diagnoses (which is not stipulated for them in clinical guidelines). Moderate sensitivity of cytology as a reference method may lead to the presence of missed severe lesions in this group. Even in our small study population, there were three patients with histological diagnoses of HSIL and CC, but a negative cytology result (NILM), which, however, were correctly assigned by our classifier to group ≥HSIL.

It is also important to mention that a number of factors, not all of which we were able to account for, may directly or indirectly influence mRNA expression in cervical epithelial cells. This may, to a greater or lesser extent, compromise the usefulness of any selected biomarkers. E.g., age-related changes have been documented for both transcripts (p16^INK4a^ and p14^ARF^) encoded by our key biomarker *CDKN2A* (55). Co-infections, age, and inflammation are themselves factors that influence carcinogenesis. Moreover, they manifest cross-effects (age affects the peculiarity of the course of infection, which is directly related to inflammation etc). Age-related changes in the transformation zone can affect the characteristics of the course of HPV infection (56), being in complex interactions with the activity of representatives of the cervical microbiome (57). The menstrual cycle significantly affects the cervical transcriptome (58). Furthermore, *MAL* and *TMPRSS4* were noted in this work as differentially expressed genes in the late secretory phase. However, patients with this cycle phase were not included in our sample (based on inclusion criterion number 2 from subsection 2.2). Another possible problem is that a cervical smear may contain not only epithelial cells but also immune system cells expressing some mRNAs from a selected set, which, depending on the level of immune infiltration, may affect their integral assessments by a molecular test. However, for all the mRNAs we selected, cancer-related expression changes have been confirmed in several studies, including the current one. Further clinical validation of the developed method on larger sample sets will allow more accurate conclusions to be drawn about the influence of the above-mentioned or other factors on the accuracy of the molecular testing result.

Thus, in this paper, we propose a method for effective detection of cervical lesions at a ≥HSIL stage (ROC AUC 0.935, sensitivity 89.7%, specificity 87.6%) via an RT-qPCR assay of five cellular mRNAs with oppositely deregulated expression in cytological preparations. Among its potential advantages are high throughput of the technique and convenience, resulting from multiplex analysis of targets and the use of lyophilized ready-for-use PCR mixes, adaptability to various clinical tasks (diagnostic strategies) owing to the adjustable cutoff for the analyzed semiquantitative characteristics, as well as the ability to conduct the analysis in parallel with HPV testing (in the same laboratory, by the same operator, on a single instrument and reagent base). Such a test can be useful at different stages of cervical examination, both in primary screening (in combination with cytology, to confirm its results, or with HPV testing, for risk stratification) and as an auxiliary tool for additional examinations in intermediate and controversial cases (LSIL, HSIL, and probably ASCUS), in well-equipped laboratories where RT-qPCR is available. In the future, for introduction into clinical practice, the method requires i) extended validation within a clinical study on a large number of cytological smears with different diagnoses (including ASCUS) as well as ii) resolving issues related to the heterogeneity of cells in cytological smears and identifying the gray zone.

## Data Availability

The original contributions presented in the study are included in the article/supplementary material. Further inquiries can be directed to the corresponding authors.
